# Schizophrenia and Violence: Systematic Review and Meta-Analysis

**DOI:** 10.1371/journal.pmed.1000120

**Published:** 2009-08-11

**Authors:** Seena Fazel, Gautam Gulati, Louise Linsell, John R. Geddes, Martin Grann

**Affiliations:** 1 Department of Psychiatry, University of Oxford, Warneford Hospital, Oxford, United Kingdom; 2 Centre for Violence Prevention, Karolinska Institute, Stockholm, Sweden & Prison and Probation Service Head Office, Norrkoping, Sweden; University of Queensland, Australia

## Abstract

**Background:**

Although expert opinion has asserted that there is an increased risk of violence in individuals with schizophrenia and other psychoses, there is substantial heterogeneity between studies reporting risk of violence, and uncertainty over the causes of this heterogeneity. We undertook a systematic review of studies that report on associations between violence and schizophrenia and other psychoses. In addition, we conducted a systematic review of investigations that reported on risk of homicide in individuals with schizophrenia and other psychoses.

**Methods and Findings:**

Bibliographic databases and reference lists were searched from 1970 to February 2009 for studies that reported on risks of interpersonal violence and/or violent criminality in individuals with schizophrenia and other psychoses compared with general population samples. These data were meta-analysed and odds ratios (ORs) were pooled using random-effects models. Ten demographic and clinical variables were extracted from each study to test for any observed heterogeneity in the risk estimates. We identified 20 individual studies reporting data from 18,423 individuals with schizophrenia and other psychoses. In men, ORs for the comparison of violence in those with schizophrenia and other psychoses with those without mental disorders varied from 1 to 7 with substantial heterogeneity (*I* ^2^ = 86%). In women, ORs ranged from 4 to 29 with substantial heterogeneity (*I* ^2^ = 85%). The effect of comorbid substance abuse was marked with the random-effects ORs of 2.1 (95% confidence interval [CI] 1.7–2.7) without comorbidity, and an OR of 8.9 (95% CI 5.4–14.7) with comorbidity (*p*<0.001 on metaregression). Risk estimates of violence in individuals with substance abuse (but without psychosis) were similar to those in individuals with psychosis with substance abuse comorbidity, and higher than all studies with psychosis irrespective of comorbidity. Choice of outcome measure, whether the sample was diagnosed with schizophrenia or with nonschizophrenic psychoses, study location, or study period were not significantly associated with risk estimates on subgroup or metaregression analysis. Further research is necessary to establish whether longitudinal designs were associated with lower risk estimates. The risk for homicide was increased in individuals with psychosis (with and without comorbid substance abuse) compared with general population controls (random-effects OR = 19.5, 95% CI 14.7–25.8).

**Conclusions:**

Schizophrenia and other psychoses are associated with violence and violent offending, particularly homicide. However, most of the excess risk appears to be mediated by substance abuse comorbidity. The risk in these patients with comorbidity is similar to that for substance abuse without psychosis. Public health strategies for violence reduction could consider focusing on the primary and secondary prevention of substance abuse.

*Please see later in the article for Editors' Summary*

## Abstract 2

Seena Fazel and colleagues investigate the association between schizophrenia and other psychoses and violence and violent offending, and show that the increased risk appears to be partly mediated by substance abuse comorbidity.

## Editors' Summary

**Background:**

Schizophrenia is a lifelong, severe psychotic condition. One in 100 people will have at least one episode of schizophrenia during their lifetime. Symptoms include delusions (for example, patients believe that someone is plotting against them) and hallucinations (hearing or seeing things that are not there). In men, schizophrenia usually starts in the late teens or early 20s; women tend to develop schizophrenia a little later. The causes of schizophrenia include genetic predisposition, obstetric complications, illegal drug use (substance abuse), and experiencing traumatic life events. The condition can be treated with a combination of antipsychotic drugs and supportive therapy; hospitalization may be necessary in very serious cases to prevent self harm. Many people with schizophrenia improve sufficiently after treatment to lead satisfying lives although some patients need lifelong support and supervision.

**Why Was This Study Done?:**

Some people believe that schizophrenia and other psychoses are associated with violence, a perception that is often reinforced by news reports and that contributes to the stigma associated with mental illness. However, mental health advocacy groups and many mental health clinicians argue that it is a myth that people with mental health problems are violent. Several large, population-based studies have examined this disputed relationship. But, although some studies found no increased risk of violence among patients with schizophrenia compared with the general population, others found a marked increase in violent offending in patients with schizophrenia. Here, the researchers try to resolve this variation (“heterogeneity”) in the conclusions reached in different studies by doing a systematic review (a study that uses predefined search criteria to identify all the research on a specific topic) and a meta-analysis (a statistical method for combining the results of several studies) of the literature on associations between violence and schizophrenia and other psychoses. They also explored the relationship between substance abuse and violence.

**What Did the Researchers Do and Find?:**

By systematically searching bibliographic databases and reference lists, the researchers identified 20 studies that compared the risk of violence in people with schizophrenia and other psychoses and the risk of violence in the general population. They then used a “random effects model” (a statistical technique that allows for heterogeneity between studies) to investigate the association between schizophrenia and violence. For men with schizophrenia or other psychoses, the pooled odds ratio (OR) from the relevant studies (which showed moderate heterogeneity) was 4.7, which was reduced to 3.8 once adjustment was made for socio-economic factors. That is, a man with schizophrenia was four to five times as likely to commit a violent act as a man in the general population. For women, the equivalent pooled OR was 8.2 but there was a much greater variation between the ORs in the individual studies than in the studies that involved men. The researchers then used “meta-regression” to investigate the heterogeneity between the studies. This analysis suggested that none of the study characteristics examined apart from co-occurring substance abuse could have caused the variation between the studies. Importantly the authors found that risk estimates of violence in people with substance abuse but no psychosis were similar to those in people with substance abuse and psychosis and higher than those in people with psychosis alone. Finally, although people with schizophrenia were nearly 20 times more likely to have committed murder than people in the general population, only one in 300 people with schizophrenia had killed someone, a similar risk to that seen in people with substance abuse.

**What Do These Findings Mean?:**

These findings indicate that schizophrenia and other psychoses are associated with violence but that the association is strongest in people with substance abuse and most of the excess risk of violence associated with schizophrenia and other psychoses is mediated by substance abuse. However, the increased risk in patients with comorbidity was similar to that in substance abuse without psychosis. A potential implication of this finding is that violence reduction strategies that focus on preventing substance abuse among both the general population and among people with psychoses might be more successful than strategies that solely target people with mental illnesses. However, the quality of the individual studies included in this meta-analysis limits the strength of its conclusions and more research into the association between schizophrenia, substance abuse, and violence would assist in clarifying how and if strategies for violence reduction are changed.

**Additional Information:**

Please access these Web sites via the online version of this summary at http://dx.doi.org/10.1371/journal.pmed.1000120.

- The US National Institute of Mental Health provides information about schizophrenia (in English and Spanish)
- The UK National Health Service Choices Web site has information for patients and carers about schizophrenia
- The MedlinePlus Encyclopedia has a page on schizophrenia; MedlinePlus provides links to other sources of information on schizophrenia and on psychotic disorders (in English and Spanish)
- The Schizophrenia and Related Disorders Alliance of America provides information and support for people with schizophrenia and their families
- The time to change Web site provides information about an English campaign to reduce the stigma associated with mental illness
- The Schizophrenia Research Forum provides updated research news and commentaries for the scientific community

## Introduction

In the 1980s, expert opinion suggested that there was no increased risk for violence in individuals with schizophrenia and other psychoses [1]. However, with the publication of large population-based studies over the last two decades, it is now thought that there is a modest association between violence and schizophrenia and other psychoses [2]. This view is not shared by many mental health clinicians [3] or public advocacy groups. For example, a recent joint public education campaign by three leading UK mental health charities contends that the view that people with mental health problems are violent is a myth [4], and the National Alliance on Mental Illness in the US asserts that acts of violence by the mentally ill are “exceptional” [5]. In factsheets, the Schizophrenia and Related Disorders Alliance of America states that people with schizophrenia are no more likely to be violent than their neighbours [6], and SANE Australia state that people with mental illness who receive treatment are no more violent than others [7]. The issue remains topical because it is thought to have contributed to policy and legal developments for psychiatric patients [8] and the striking increase in the number of secure hospital patients in many Western countries (alongside sex offender legislation) [9]. It also contributes to the stigma associated with mental illness [10], which is considered to be the most significant obstacle to the development of mental health services [11].

Although there have been a number of studies examining the relationship between the psychoses and violent outcomes, there are wide variations in risk ratios reported with estimates ranging from 7-fold increases in violent offending in schizophrenia compared with general population controls [12],[13] to no association in a highly influential prospective investigation [14]. Previous reviews of the literature have not been quantitative or have not systematically explored the grey literature [9]–[12]. In addition, they have included selected samples, such as investigations solely of homicide offenders (who are more likely to have psychoses than other offenders) [15], and have not explored potential sources of heterogeneity.

We report a systematic review of investigations examining the risk of schizophrenia and other psychoses for violent outcomes including homicide. We explored the reasons for variations between the primary studies using metaregression. We aimed to test whether risk estimates differed by gender, diagnosis (schizophrenia versus other psychoses), outcome measure (criminal convictions versus self-report or informant based information), country location (US or Nordic countries versus the rest of the world), study design (case-control versus longitudinal), and study period. In addition, we have conducted a systematic review of studies examining the risk of schizophrenia in homicide offenders.

## Methods

Computerised Medline, Embase, and Psycinfo searches were performed from January 1970 to February 2009 using the terms viol*, crim*, homicide, schiz*, severe mental illness, major mental disorder, psychos*, and psychot*. References were hand searched for other references, including to grey literature, and non-English language publications were translated. In order to supplement the search of grey literature, US National Criminal Abstracts was searched as well as an extensive bibliography on crime and mental disorder prepared for the Public Health Agency of Canada [16]. We contacted authors of published studies for additional information as required. MOOSE guidelines (Meta-analyses of Observational Studies in Epidemiology, http://www.consort-statement.org/index.aspx?o=1031) were followed.

Our inclusion criteria included case-control studies (including cross-sectional surveys) and cohort studies, which allowed an estimation of the risk of violence in patients with schizophrenia and/or other psychoses compared with a general population comparison group.

Reports were excluded if: (i) Data were presented solely on all convictions not broken down for violence [17]. (ii) There was no general population comparison data [18]–[20]. Studies that used other psychiatric diagnoses as the comparator group were also excluded [21]. (iii) Data were superseded by subsequent work and inclusion would involve duplication of data [13],[22]–[25]. In one of these studies [24], data were used for the subgroup analysis on whether outcomes were different by diagnosis of cases (schizophrenia versus nonschizophrenic psychoses). In another, data for women were used from the older publication because it was not included in the updated work [25]. (iv) The cases included diagnoses of nonpsychotic illnesses such as personality disorder [14] and major depression [26]. However, we included one study where the proportion of psychoses was 95% [27].

We conducted a separate analysis of homicide only studies. For this analysis, studies were excluded if information on controls was taken from a different country and another time period [28],[29] or no data on controls were provided [30],[31]. For one of the included studies [32], state population data were specifically gathered from a government agency [33], and for another [24], data on homicides were specifically extracted for the purposes of this review.

### Data Extraction

A standardised form was used to extract data, which included information on the study design, geographical location of study, last year of follow-up for violence (“study period”), diagnoses of cases, definition of violence, method of ascertainment of violence, sample size, mean age, adjustment for socio-demographic factors, and, in the cases, numbers with comorbid substance abuse. For those studies with comorbid substance abuse data, we also extracted data on primary and secondary diagnoses of substance abuse in the population controls (and in two comparisons [24],[34], these were extracted from data based on separate publications [25],[35]). Where possible, the control group was a population of individuals without any mental disorders. If data were available for both schizophrenia and nonschizophrenic psychoses, the former was used for the primary analyses.

For the purposes of analysis, study design was explored as a dichotomous variable (case-control versus longitudinal) where nested case-control study were included as case-control designs, and also all three designs were compared (case-control versus nested case-control versus longitudinal). Longitudinal designs referred to studies where violence was assessed after diagnosis had been established. Study location was analyzed in two ways: Nordic countries versus the rest of the world, and the US versus the rest of the world. The analysis was done in this way because many of the studies were conducted in three Nordic countries (Sweden, Denmark, Finland) because of the availability for research of national registers for health and crime, and the possibility that the gun ownership laws and higher base rates of violence in the US lead to different risk estimates than other countries [36]. Sample size was analyzed as a continuous variable (for the metaregression) and by numbers of cases in three groups (0–99, 100–1,000, and >1,001 cases) for subgroup analysis. Outcomes measures were analyzed as a dichotomous variable: register-based versus self-report and/or informant interview. Study period was assessed by those reports where last year of follow-up was before 1990 and those on or after 1990. Gender was included in the metaregression analysis as a trichotomous (male, mixed, and female studies separately) and dichotomous (male and mixed studies combined versus female) variable.

Suitability for inclusion was assessed and data extraction conducted independently by two researchers (SF and GG), and any differences resolved with discussion with the other authors.

### Data Analyses

Meta-analyses of risk of violent outcomes were carried out generating pooled odds ratios (ORs) with 95% confidence intervals (CIs). Heterogeneity among studies was estimated using Cochran's *Q* (reported with a χ^2^-value and *p*-value) and the *I* ^2^ statistic, the latter describing the percentage of variation across studies that is due to heterogeneity rather than chance [37],[38], with 95% CIs [38]. *I* ^2^, unlike *Q*, does not inherently depend upon the number of studies considered with values of 25%, 50%, and 75% taken to indicate low, moderate, and high levels of heterogeneity, respectively.

We explored the risk associated with substance abuse comorbidity separately by presenting estimates of risk ratios of schizophrenia and related psychoses with comorbidity, and without comorbidity. As others have noted, adjustment by substance abuse is not appropriate as it exists on the causal pathway between schizophrenia (exposure) and outcome (violence) [39],[40]. We calculated adjusted ORs by socio-demographic factors when stratum-specific estimates were given using the Mantel-Haenszel method [41].

We calculated population attributable risk fractions for the studies that reported on number of crimes in the samples investigated. We opted for individual counts of crime rather than number of convicted individuals for this analysis as it has been demonstrated that the number of crimes per conviction is significantly higher in individuals with severe mental illness than other offenders [24]. Hence, using crimes more accurately captures the population impact of violent criminality. For this analysis, the base rate *r* was defined as the number of separate violent crimes committed per 1,000 in the general population. *r*_0_ was defined as the number of violent crimes per 1,000 individuals who had not been patients with schizophrenia. We then calculated the population-attributable risk as the difference in *r*−*r*_0_ and the population-attributable risk fraction as population-attributable risk/*r*. These data were not synthesized because of their heterogeneity.

Potential sources of heterogeneity were investigated further by metaregression analysis, subgrouping studies according to their inclusion criteria, and methodological factors. All subgroup analyses involved nonoverlapping data and used random-effects models. For metaregression analyses, male, female, and mixed-gender studies were included. All factors were entered individually and in combination to test for possible associations. Analyses were done in STATA statistical software package, version 10 (Statacorp, 2008) using the metan (for random and fixed-effects meta-analysis), metareg (for metaregression), and metabias (for publication bias analysis).

## Results

Twenty individual studies were identified (for details of the studies see Table S1). The total number of schizophrenia and other psychoses cases in the included studies was 18,423. Of these cases, 1,832 (9.9%) were violent. These cases were compared with 1,714,904 individuals in the general population, of whom 27,185 (1.6%) were violent. Publications were from 11 countries: five from the US (874 cases, 4.7% of total number of cases) [42]–[46]; two from England and Wales (*n* = 66, 0.4%) [27],[47]; two from Denmark (*n* = 1,873, 10.2%) [48],[49]; three from Sweden (*n* = 9,024, 49.0%) [50]–[52]; two from Finland (*n* = 90, 0.5%) reported in three publications [12],[53],[54]; one from Australia (*n* = 2,861, 15.5%) [55]; Germany (*n* = 1,662, 9.0%) [56]; Austria (*n* = 1,325, 7.2%) [57]; Switzerland (*n* = 508, 2.8%) reported in three publications [25],[34],[58]; New Zealand (*n* = 39, 0.2%) [59]; and Israel (*n* = 101, 0.5%) reported in two publications [60],[61]. Violence was ascertained from register-based sources in 13 studies, by self-report and informants in five others, and in two investigations by both methods [43],[59].

### Male Studies

**Figure 1:**
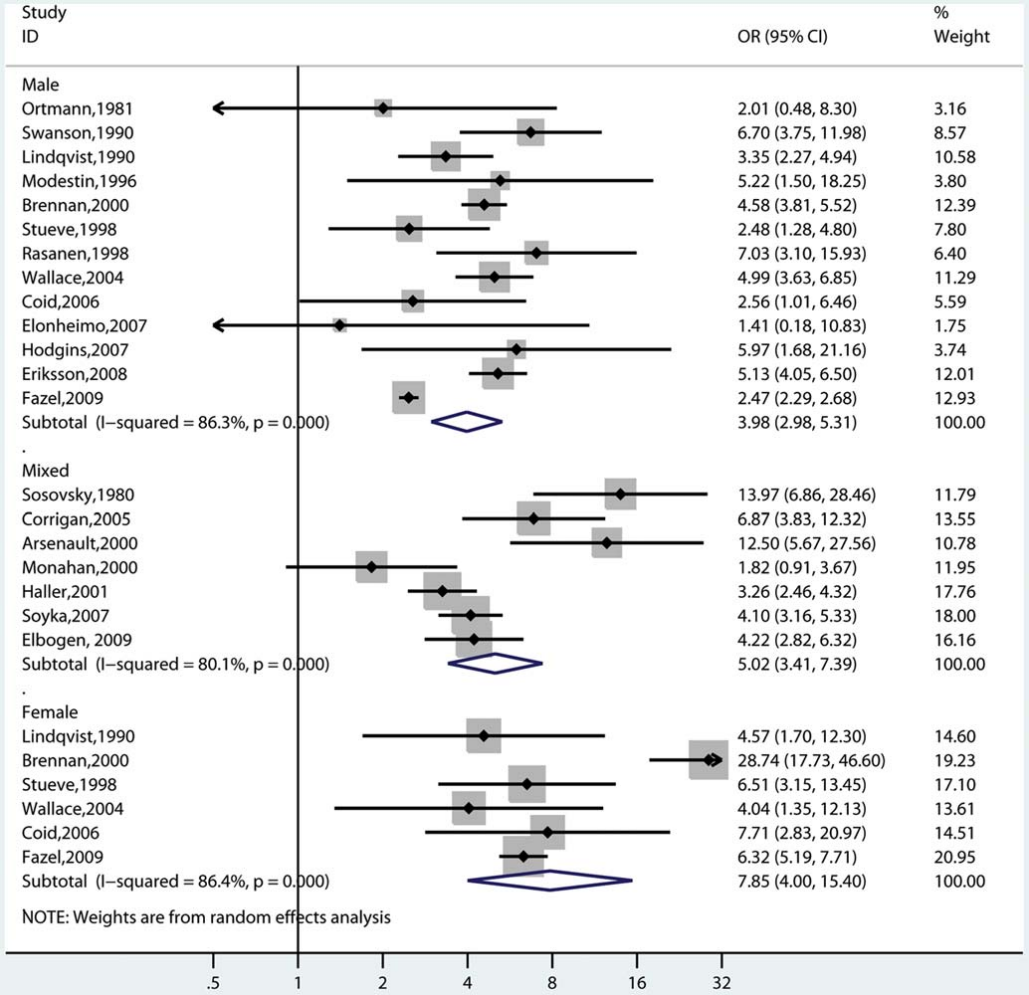
Risk estimates for violence in schizophrenia and other psychoses by gender. Note: Mixed refers to studies where both genders have been included. These estimates are for ORs that are mostly not adjusted for socio-economic factors although Monahan and Wallace have matched cases and controls by neighbourhood of residence and Modestin for occupational level and marital status (see Table S1).

In the men, 13 studies were identified with 9,379 individuals with schizophrenia and other psychoses (Figure 1) [27],[45],[47]–[55],[58],[61]. The random-effects pooled crude OR comparing the risk of violence in cases with general population controls was 4.0 (95% CI 3.0–5.3) with substantial heterogeneity (*I* ^2^ = 88%, 95% CI 78–91). When using fixed-effects models, the overall crude OR for men was 2.9 (95% CI 2.7–3.1). When adjusting for socio-economic factors, possible in four of these studies [12],[49],[52],[60], the random-effects OR was 3.8 (2.6–5.0), and fixed-effects OR was 2.0 (1.8–2.1) with high heterogeneity (*I* ^2^ = 84% [74%–90%]).

### Female Studies

Six studies provided risk estimates in female samples in 5,002 individuals with schizophrenia and other psychoses (Figure 1) [47],[49],[51],[52],[55],[61]. The random-effects pooled crude OR was 7.9 (95% CI 4.0–15.4), and the fixed-effects crude OR was 6.6 (5.6–8.0). These estimates were associated with high heterogeneity (*I* ^2^ = 86% [73%–93%]. Three additional studies that included 256 women with schizophrenia made no material difference to the risk estimates (random-effects pooled OR = 7.7; 4.2–14.1) [12],[25],[27]. These studies were excluded from sensitivity analyses as the base rate of violent was zero in the cases [27],[53] or the controls [25], and thus led to unstable risk estimates.

### Mixed Gender Studies

Seven studies reported risk of violence in mixed samples (*n* = 3,786, 20.6% of all cases) [42]–[44],[46],[57],[59],[62], which reported an increased risk of violence compared with general population controls. The random-effects pooled OR was 5.0 (3.4–7.4), and the fixed-effects OR was 4.0 (3.4–4.7) with an *I* ^2^ of 80% (59%–90%).

### Substance Abuse Comorbidity

**Figure 2:**
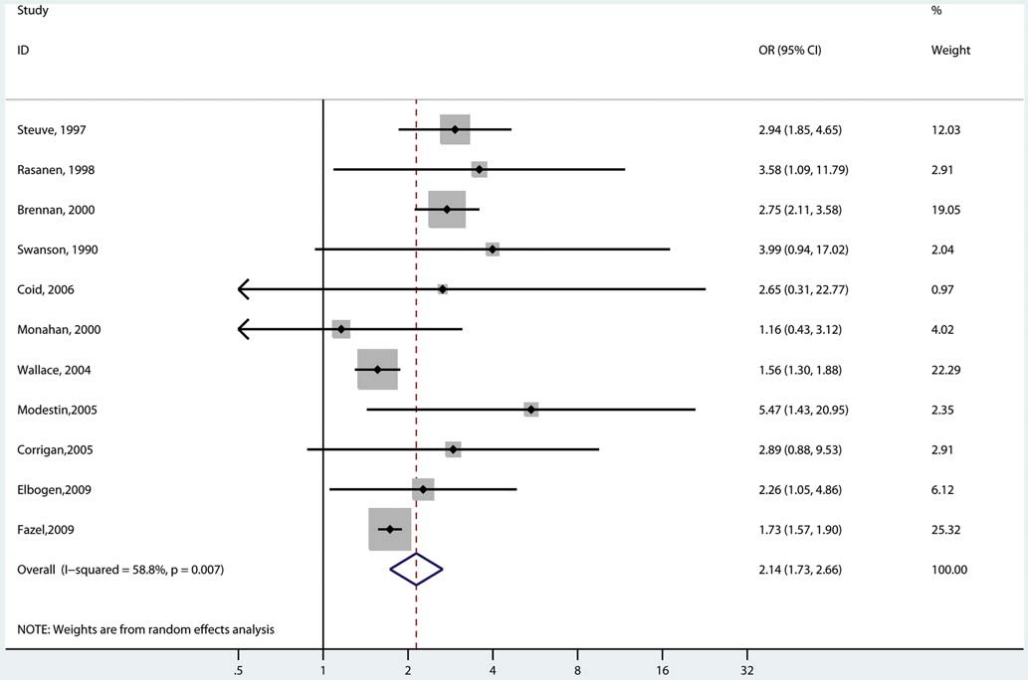
Risk estimates for violence in schizophrenia and other psychoses with no substance abuse comorbidity.

**Figure 3:**
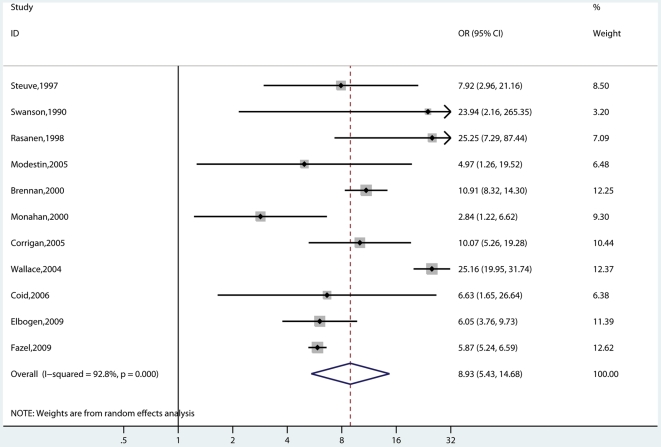
Risk estimates for violence in schizophrenia and other psychoses with substance abuse comorbidity.

Eleven studies involving 2,891 cases reported on risk of violence with and without substance abuse (Figures 2 and 3) [34],[42]–[45],[47],[49],[52],[53],[55],[60]. In six of these studies [42]–[44],[47],[52],[55], these were mixed gender samples. Risk of violence was raised in individuals of any gender with psychosis and comorbidity (random-effects OR = 8.9; 5.4–14.7; *I* ^2^ = 93%; 89%–95%) compared with general population controls. Violence risk was lower in persons with psychosis without comorbidity (OR = 2.1; 1.7–2.7; *I* ^2^ = 59%; 19%–79%) in comparison with general population controls. When this analysis was confined to the five studies that reported in men [34],[45],[49],[53],[55], the OR without comorbidity was 2.8 (2.3–3.5) (*I* ^2^ = 0%; 0%–66%) compared with an OR with comorbidity of 12.2 (9.5–15.8) (*I* ^2^ = 13%; 0%–57%). One study reported risk estimates in women with schizophrenia [49]. The OR without comorbidity was 19.9 (10.7–36.8), and with comorbidity, it was 74.8 (35.8–156.1). Substance abuse was highly significant on metaregression (β = −1.35, standard error [SE] = 0.26, *t* = −5.24, *p*<0.001).

### Heterogeneity

**Figure 4:**
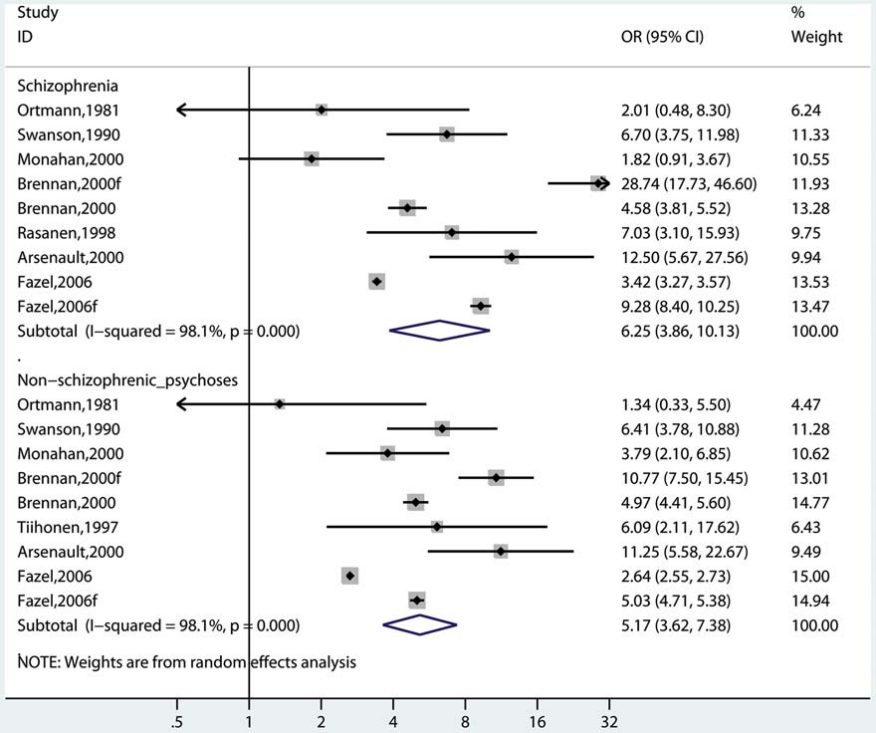
Risk estimates for violence by diagnosis of cases in studies that reported both diagnoses.

We examined possible differences between risk estimates by various characteristics (Table 1). There was no significant difference in the risk estimates by diagnostic criteria of the cases. This result was demonstrated in three different ways. When including all the studies, the OR for risk of violence in individuals with schizophrenia was 5.6 (4.1–7.6) compared with the risk in nonschizophrenia psychoses where the OR was 4.9 (3.6–6.6). When this analysis was limited to those studies that reported both diagnoses, there was no difference (OR = 6.3, 3.9–10.1 in schizophrenia versus OR = 5.2, 3.6–7.4 in nonschizophrenia psychoses) (Figure 4). When it was limited to men and those that reported both diagnoses, those with schizophrenia had a risk estimate of 4.0 (3.0–5.3), similar to individuals with nonschizophrenia psychoses, where the OR was 4.0 (3.3–4.8).

**Table 1:** Risk estimates for possible sources of heterogeneity in studies of violence in individual with schizophrenia and other psychoses.

Sample or Study Characteristics	*n* Studies	*n* Cases	OR (95% CI)	Heterogeneity *I* ^2^ (95% CI)
Type of psychosis				
Schizophrenia^a^	13	8,578	5.5 (4.1–7.5)	96 (95–97)
Nonschizophrenia psychoses	7	99,668	4.9 (3.6–6.6)	97 (96–98)
Outcome				
As arrest or conviction^b^	14	17,312	5.3 (3.9–7.2)	93 (90–95)
As self-report	7	894	4.9 (3.2–7.3)	70 (39–85)
Study				
Based in Scandinavia	7	10,962	5.0 (3.2–7.8)	96 (94–97)
Based outside of Scandinavia	13	7,205	4.8 (3.8–6.1)	62 (34–78)
Based in US	5	874	5.5 (3.0–9.8)	80 (53–92)
Based outside of US	15	17,293	4.9 (3.7–6.4)	91 (88–94)
Conducted before 1990	5	3,084	6.6 (3.6–11.8)	90 (82–94)
Conducted in 1990 or after	15	15,083	4.9 (4.2–5.7)	89 (84–92)
Design				
Case-control	11	7,356	4.7 (3.8–5.8)	51 (9–72)
Nested case-control	6	2,354	6.9 (3.9–12.2)	90 (81–94)
Longitudinal (cohort)	3	8,457	3.4 (1.9–6.3)	96 (93–98)
*n* Cases				
<100^c^	12	2,577	5.3 (3.1–9.0)	83 (73–90)
= 101–1,000	6	4,528	5.3 (4.0–6.9)	65 (21–84)
>1,000	5	11,062	3.7 (2.7–5.2)	93 (87–96)

a Number of cases differs in this analysis because data were included from Fazel [24].

b Number of studies and cases differs because data from Arseneault [59] contribute to both cells.

c Number of studies is >20 because three studies contributed to more than one cell as the number of cases of the male and female samples differs for Brennan [49], Fazel [52], and Wallace [55].

**Figure 5:**
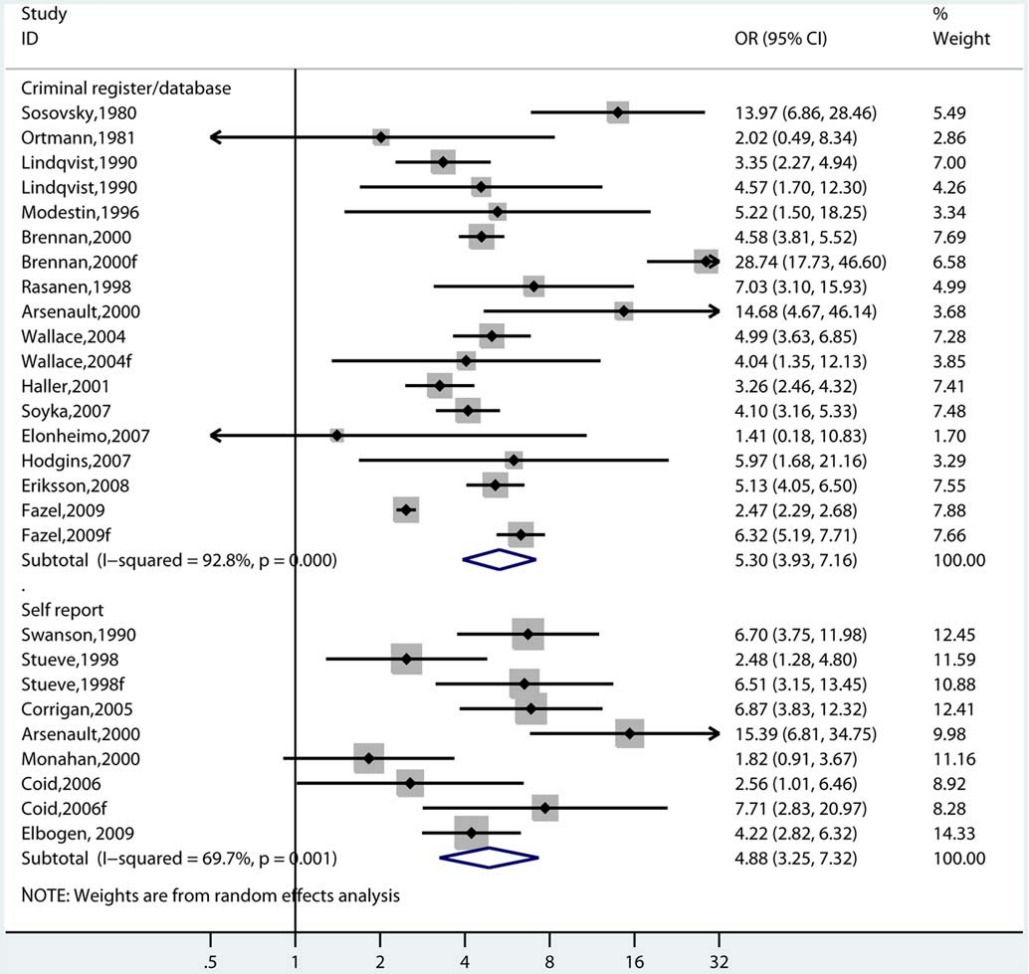
Risk estimates for violence in schizophrenia and other psychoses by outcome measure. Note: Self-report also includes informant-based sources.

There was no difference in risk estimates depending on type of outcome measure (criminal convictions and arrest data versus self- and informant-report; Figure 5). In the male-only studies, the OR was 4.1 (3.0–5.5) for the register-based outcomes, whereas it was 3.0 (1.6–5.8) for the investigations where self-report and informants were used to determine outcome. These were both associated with substantial heterogeneity (*I* ^2^'s of 88% and 70%, respectively). There was only one study where risk estimates on both outcomes were reported [59].

**Figure 6:**
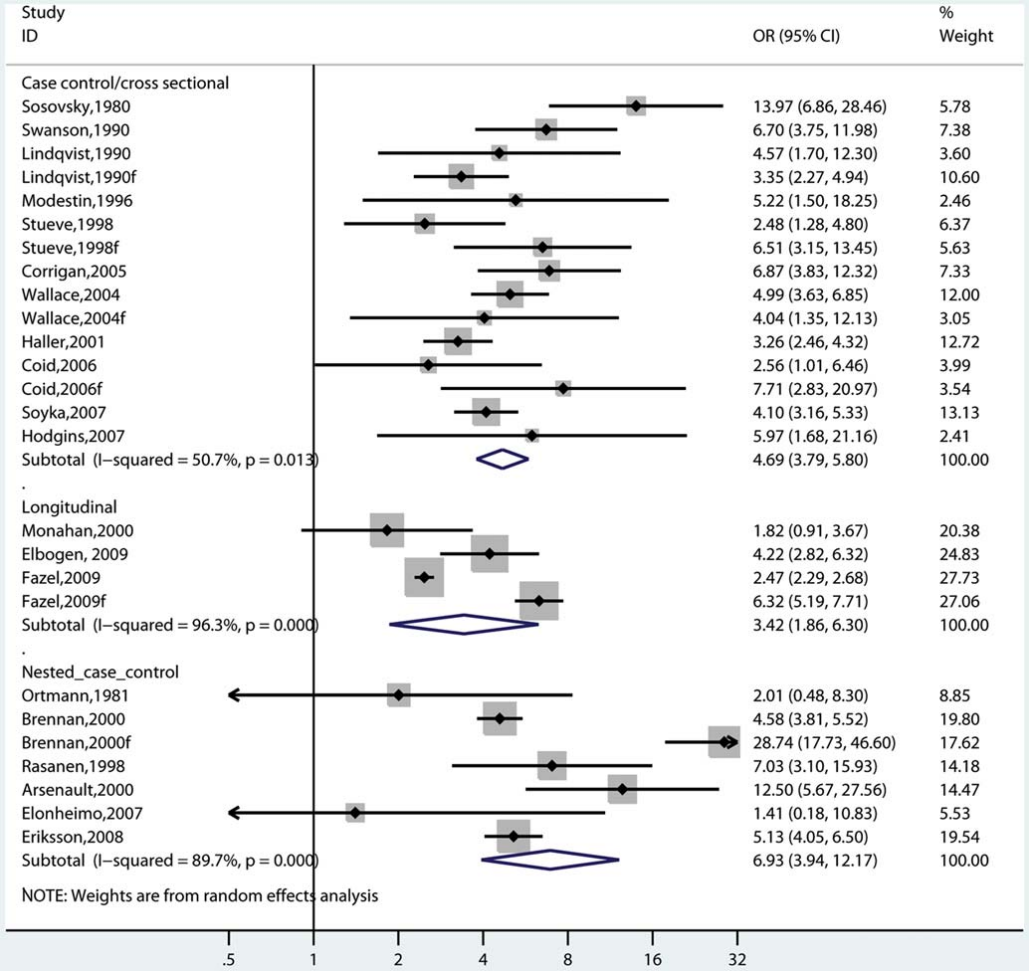
Risk estimates for violence in schizophrenia and other psychoses by study design.

There was no evidence of any difference in risk estimates by region when comparing studies conducted in Nordic reports with those from other countries (Table 1), or when the studies based in the US were compared with the rest of the world. In the male-only studies, the Nordic ones reported an OR of 4.4 (3.5–5.4) compared with the rest of the world where the OR was 3.8 (2.6–5.5). There was no significant difference in risk estimates for the other study characteristics: study period and study size. Nonsignificant differences by study type were found: longitudinal studies reported lower risk estimates (OR = 3.8, 2.6–5.5) but this was based on only four samples (Figure 6). Furthermore, there was some evidence of publication bias using Egger's test (*t* = 2.17, *p* = 0.04) but not with a funnel plot analysis (*z* = −0.31, *p* = 0.76). This finding was replicated when we combined the results for gender and used the publication as the unit of measurement (Egger's test, *t* = 2.75, *p* = 0.013; funnel plot, *z* = −0.39, *p* = 0.70).

In the metaregression analysis with all the studies included, none of these study characteristics apart from substance abuse was statistically significant (individually or in a model where all factors were entered into simultaneously). Study type as a dichotomous variable (longitudinal versus case-control) was associated with some heterogeneity on metaregression when all factors were included in the model (β = −1.12, *t* = −1.57, *p* = 0.12). When the analysis was restricted to the male and mixed gender studies, the association almost reached statistical significance (β = −1.64, *t* = −2.44, *p* = 0.051).

### Substance Abuse and Violent Crime

**Figure 7:**
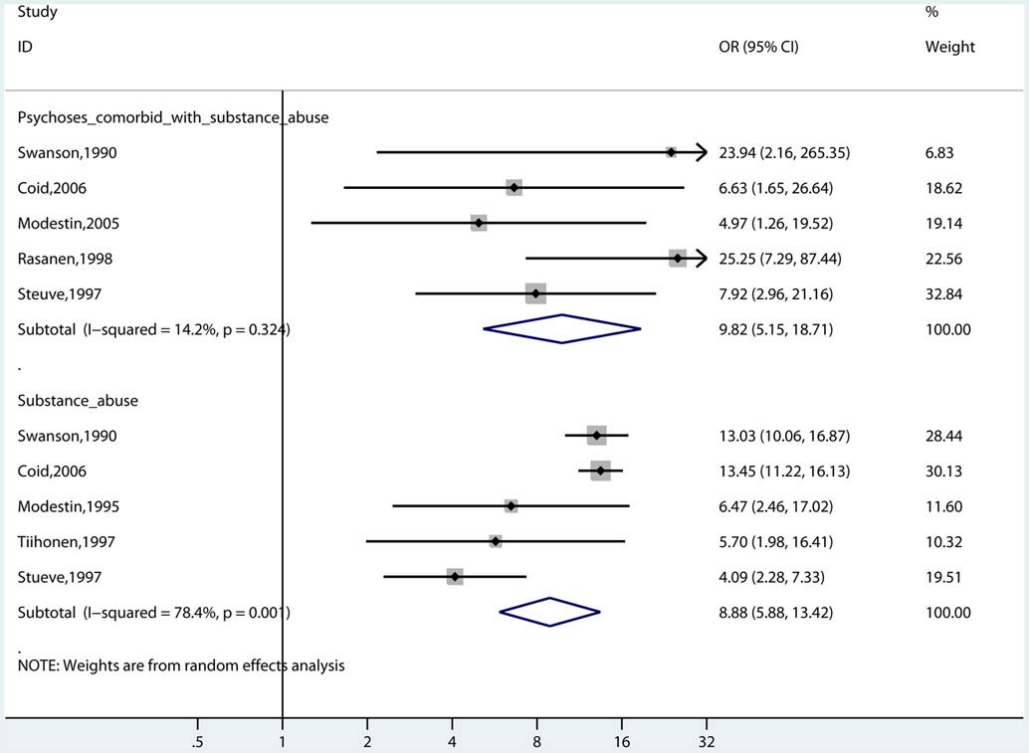
Risk estimates for violence in men with schizophrenia comorbid with substance abuse compared with risk in men with substance abuse (without psychosis) reported in the same study.

**Figure 8:**
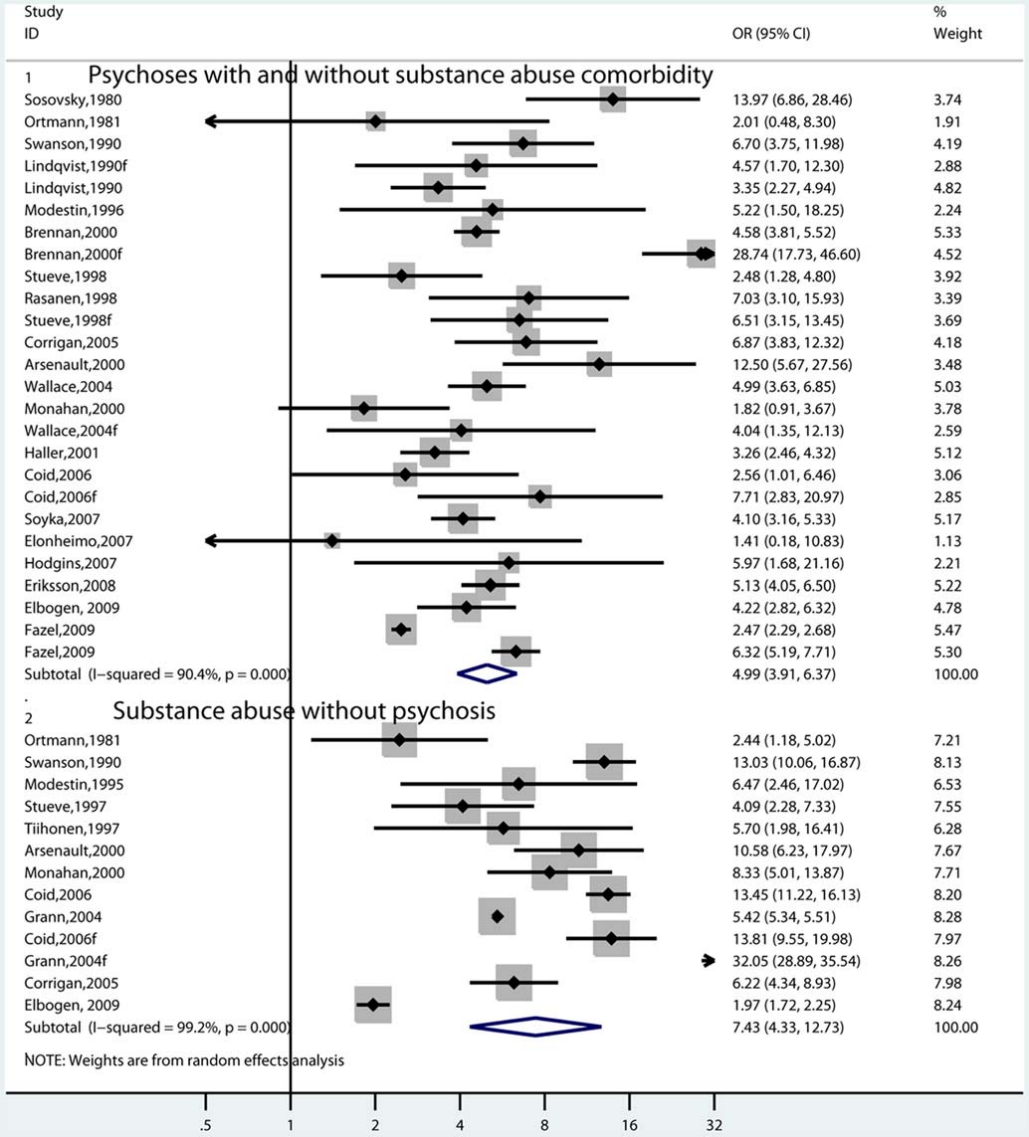
Risk estimates for violence in schizophrenia and other psychoses compared with risk in individuals with substance abuse. Note: Psychoses studies include individuals with psychotic disorders of both genders with and without substance abuse comorbidity. Substance abuse studies involve risk estimates of violence in individuals of both genders with a diagnosis of substance abuse.

In men, there were five studies where the risk of violence was reported both in individuals with schizophrenia and other psychoses who have comorbid substance abuse, and in individuals with substance abuse alone [12],[25],[34],[45],[47],[53],[61]. In comparing these risk estimates, there was no apparent difference (Figure 7). We also compared all psychoses studies (irrespective of comorbidity) with those that reported risk of violence in individuals with a diagnosis of substance use disorders (Figure 8). Substance use disorders were associated with higher risk estimates, although the finding was nonsignificant using a random-effects model. Using fixed-effects, the OR in individuals with psychosis was 3.3 (3.0–3.5) compared with 5.5 (5.4–5.6) in substance abuse.

### Population Attributable Risk Fractions

We identified six studies where population attributable risk fractions could be extracted because information on the number of crimes was presented in addition to the number of convicted persons. These were no more than 10%, and were: 3.2% [55], 3.5% [50], 5.2% [24], 8.2% [54], 8.4% [59], and 9.9% [49].

### Homicide as Outcome

**Figure 9:**
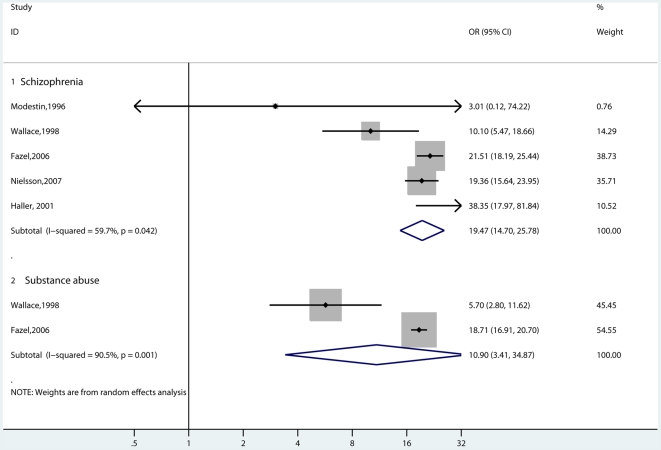
Risk estimates for homicide in individuals with schizophrenia and in individuals with substance abuse.

We identified five studies that reported on the risk of homicide in individuals with psychosis compared with the general population (Figure 9) [23],[24],[32],[57],[58]. There were 261 homicides committed by individuals with schizophrenia and other psychoses compared with 2,999 in the comparison group. The risk of homicide in individuals with schizophrenia was 0.3% compared with 0.02% in the general population. The random-effects pooled OR was 19.5 (14.7–25.8), with significant heterogeneity (*I* ^2^ = 60%; 0%–85%). Within these studies, we compared these estimates with the two studies that reported on risk of homicide in persons diagnosed with substance abuse. The risk of homicide in individuals with substance abuse was 0.3%, with a random-effects pooled OR of 10.9 (3.4–34.9).

## Discussion

This systematic review of the risk of violence in schizophrenia and other psychoses identified 20 studies including 18,423 individuals with these disorders. There were four main findings. The first was that the risk of violent outcomes was increased in individuals with schizophrenia and other psychoses. The risk estimates, reported as ORs, were all above one indicating an increased risk of violence in those with schizophrenia and other psychoses compared with the general population controls, although the risk estimates varied between one and seven in men, and between four and 29 in women. A second finding was that comorbidity with substance use disorders substantially increased this risk, with increased ORs between three and 25. Although there was considerable variation in this estimate between studies, the pooled estimate was around four times higher compared with individuals without comorbidity. Third, we found no significant differences in risk estimates for a number of study design characteristics for which there has been uncertainty. These included: whether the diagnosis was schizophrenia versus other psychoses, if the outcome measure was register-based arrests and convictions versus self-report, and if the study location was the US or Nordic countries compared with other countries. Finally, the increased risk of violence in schizophrenia and the psychoses comorbid with substance abuse was not different than the risk of violence in individuals with diagnoses of substance use disorders. In other words, schizophrenia and other psychoses did not appear to add any additional risk to that conferred by the substance abuse alone.

We found higher risk estimates in the female-only and mixed gender studies compared with the general population, although these estimates were not significantly higher than male-only estimates using random-effects models. The higher risk estimates in women may be a consequence of the lower prevalence of drug and alcohol use in the general female population compared with the general male population, and so violence associated with other causes, including schizophrenia, would be overrepresented in the women [24]. Although other work has demonstrated a closing of the gender gap in rates of violence from patients discharged from psychiatric hospitals [63], this present systematic review has shown that risk of violence by gender is reversed compared with general population prevalence rates of violence. In addition, we found only five studies that compared risk of homicide in individuals with schizophrenia compared with the general population. Although the heterogeneity was large, the risk estimates were considerably higher than those for all violent outcomes. Although the risk of any individual with schizophrenia committing homicide was very small at 0.3% and similar in magnitude to the risk in individuals with substance abuse (which was also 0.3%), it does indicate a particularly strong association of psychosis and homicide. It may also reflect the better quality of these studies, including better ascertainment of cases. Apart from homicide, risk estimates do not appear to be elevated with the increasing severity of violent offence in individuals with psychosis [24],[52].

There were several potentially important negative findings. In particular, Nordic-based or US-based investigations did not provide different risk estimates than the rest of the world. This finding would argue against the suggestion that the association between mental illness and violent crime is modified by variations in population base rates of violence [36] or the availability of handguns. Lastly, there was no difference in risk estimates produced by studies conducted before and after 1990. Although deinstitutionalization would have occurred at different dates in the included studies, this finding may support the conclusions of two related investigations in the Australian state of Victoria that demonstrated that violent convictions have not increased in recent decades compared to these offences in the general population [13],[55]. Further research is needed to examine this issue.

There are a number of limitations to this review. First, caution is warranted in the overall estimates provided in this review as there was significant heterogeneity. The lack of any explanation for this heterogeneity, apart from substance abuse comorbidity and possibly study design, suggests that methodological variations that we were not able to test may have been important. An alternative approach would be individual participant meta-analysis as it would provide some consistency across the potentially mediating characteristics. One notable finding was that, in all but three of the included studies, violence was assessed irrespective of the timing of the diagnosis of schizophrenia (i.e., violence before and after the diagnosis), which would overestimate the effects of the illness. There were three studies that used longitudinal designs (where violence was only included after diagnosis was established) [42],[43],[52], which provided lower risk estimates. Second, the overall pooled estimates will have overestimated the association because of inadequate adjustment for confounding and the use of a random-effects meta-analysis. A consequence of the latter was that risk estimates were less conservative than using a fixed-effects model, as the smaller studies were weighted more equally in the random-effects meta-analysis [64]. For example, in the men, the pooled OR was 2.9 in the fixed-effects model compared with a random-effects estimate of 4.0. The fixed-effects odd ratio was further reduced to 2.0 when adjustment for socio-demographic factors was included, possible in only four out of 13 male studies. However, the use of random-effects estimates in the subgroup analysis led to more conservative findings because of larger CIs. Another limitation was there were no studies outside of the US, Northern Europe, Israel, Australia, and New Zealand potentially limiting the generalizability of the findings. However, we found no difference by study region (such as the US or Nordic countries compared with other countries), which would suggest that the findings are applicable to Western countries. However, the lack of any studies in low income countries is notable.

A number of recommendations for future research arise from this review. Residual and inadequate confounding is likely to have affected the estimates produced by the primary studies because of inadequate measurement of exposures and confounders. For example, some of the studies adjusted for socio-economic status by using the profession of the father [53], while another used neighbourhood controls [55]. More precise and reliable measures of confounders need to be included in future studies. One promising approach is to compare individuals with schizophrenia with unaffected siblings, and there is a recent study that found that the adjusted OR of violent crime for individuals with schizophrenia compared with their unaffected siblings was 1.8 (95% CI 1.3–1.8). When compared with general population controls matched for year of birth and gender, the adjusted OR was 2.0 (1.8–2.2) [52]. In addition, how substance abuse mediates violent offending needs further study. Whether future work needs to rely on resource-intensive ways of gathering outcome data such as self-report measures or interviewing informants is questioned by this review, although prevalence rates will be higher when such approaches are used. In addition, health services research could further examine the role of different service configurations in reducing violence outcomes in these patients. In particular, the role of continuity of care should be investigated. Research has demonstrated no reduction in the prevalence of violence when intensive case management has been used compared with standard care [65], but alternative models of service delivery need study. Finally, perhaps the most important research implication is the need for better quality and larger randomized controlled trials for the treatment of substance abuse comorbidity in schizophrenia [66].

A number of implications arise from this review. First, the findings highlight the importance of risk assessment and management for patients with substance abuse comorbidity. In those without substance abuse comorbidity, the risk of violent crime was modestly elevated with ORs ranging from 1 to 5. However, better adjustment for potentially relevant confounders and problems of misclassification (i.e., many of these patients may have undiagnosed and unreported substance abuse) would possibly reduce the observed risk. This effect has been demonstrated in a recent Swedish study where the adjusted OR was minimally raised (at 1.2) in individuals with schizophrenia and no comorbid substance abuse compared with general population controls [52]. The relationship between comorbid substance abuse and violence in schizophrenia may be mediated by personality features and/or social problems, and is unlikely to be a simple additive effect [67]. In support, one study demonstrated that rates of substance abuse have increased markedly in individuals with schizophrenia over 25 y, but rates of violence modestly. The authors concluded that a subgroup of people with schizophrenia at risk of violence have increasingly abused substances [55]. The relationship with medication adherence may also mediate the association with violent outcomes, particularly if it precedes substance abuse on the causal pathway to violence. The data on medication adherence has reported associations with violence in naturalistic studies [68], but a recent analysis of the Clinical Antipsychotic Trials in Intervention Effectiveness (CATIE) trial data for violent outcomes found no overall association with violence [69]. Further research is necessary to clarify the relationship between substance abuse, medication adherence, and violence. A second implication relates to attempts to redress the stigmatization of patients with schizophrenia and other psychoses that could be reconsidered in light of the findings of the risk of violence in substance use disorders [11]. Our findings suggest that individuals with substance use disorders may be more dangerous than individuals with schizophrenia and other psychoses, and that the psychoses comorbid with substance abuse may confer no additional risk over and above the risk associated with the substance abuse. As substance use disorders are three to four times more common than the psychoses [70],[71], public health strategies to reduce violence in society could focus on the prevention and treatment of substance abuse at individual, community, and societal levels [35],[72],[73].

In summary, there is a robust body of evidence that demonstrates an association between the psychoses and violence. This association is increased by substance abuse comorbidity and may be stronger in women. However, the increased risk associated with this comorbidity is of a similar magnitude to that in individuals with substance abuse alone. This finding would suggest that violence reduction strategies could consider focusing on the primary and secondary prevention of substance abuse rather than solely target individuals with severe mental illness.

## Supporting Information

Table S1 Details of studies estimating risk of violence in individuals with schizophrenia and other psychoses.
(0.08 MB DOC)

## Abbreviations

CI: confidence interval
OR: odds ratio
