# Supplementary material for: Schizophrenia and Violence: Systematic Review and Meta-Analysis
Source: PLoS Med. 2009 Aug 11;6(8):e1000120. doi: 10.1371/journal.pmed.1000120 (PMC2718581; doi:10.1371/journal.pmed.1000120)
Supplement: Table S1 — Details of studies estimating risk of violence in individuals with schizophrenia and other psychoses. (0.08 MB DOC) [file pmed.1000120.s001.doc]

**Table S1. Details of studies estimating risk of violence in individuals with schizophrenia and other psychoses**

| **Study** | **N Scz Viol** | **N Scz** | **N Pop Viol** | **N Pop** | **Type of Study** | **Ascertainment of Violence** | **Definition of cases** | **Gender** | **Location** | **Study period** | **Definition of violence** | **Cases diagnosed by** | **Comparison Group** | **Drug abuse defined by** | **Mean Age of cases in years** |
| --- | --- | --- | --- | --- | --- | --- | --- | --- | --- | --- | --- | --- | --- | --- | --- |
| Sosovsky, 1980 | 8 | 171 | 1657 | 473471 | Case-control | Register | Schizophrenia | Mixed | US | 1975 | Violent crimes | Not stated | General population | - | - |
| Ortmann,  1981 | 2 | 50 | 234 | 11553 | Nested Case Control | Register | Schizophrenia | Male | Denmark | 1978 | Violent offences excluding sexual offences | ICD-8 | General population | - | 25 |
| Swanson,  1990 | 14 | 114 | 165 | 8066 | *Case-control | Self report | Schizophrenia | Male | US | 1983 | DIS items indicating self reported violent behaviour | Diagnostic Interview Schedule (DIS) leading to DSM-III diagnoses | General population members with No disorder | DSM III diagnoses of alcohol or drug abuse or dependence | - |
| Lindqvist, 1990 | 28 | 330 | 4173 | 154819 | Case-control | Register | Schizophrenia | Male | Sweden | 1986 | Assault, aggravated assault, petty assault, unlawful deprivation of liberty, unlawful threats, violence against officials, threat against officials | Discharge diagnosis as per ICD -8 | General Swedish population – crime data estimated from one year rates (1978). Data supplied on request from Dr Lindqvist | - | 30-39 |
| Lindqvist, 1990 | 4 | 314 | 424 | 150502 | Case-control | Register | Schizophrenia | Female | Sweden | 1986 | Assault, aggravated assault, petty assault, unlawful deprivation of liberty, unlawful threats, violence against officials, threat against officials | Discharge diagnosis as per ICD -8 | General Swedish population – crime data estimated from one year rates (1978). Data supplied on request from Dr Lindqvist | - | 30-39 |
| Modestin,  1996 | 15 | 282 | 3 | 282 | Case-control | Register | Schizophrenia | Male | Switzerland | 1987 | Violent crimes including assaults resulting in bodily harm and murder but excluding sexual offences | RDC diagnoses | General population subjects matched with the patients age, sex, marital status, occupational level and community size | ICD-9 codes: 303-305 | 39 |
| Modestin 1995 | 1 | 226 | 0 | 226 | Case-control | Register | Schizophrenia and other psychoses | Female | Switzerland | 1985-7 | Violent crimes including assaults resulting in bodily harm and murder but excluding sexual offences | RDC diagnoses | General population subjects matched with the patients age, sex, marital status, occupational level and community size |  | 44 |
| Rasanen,  1998/Tiihonen , 1997 | 7 | 51 | 117 | 5287 | Nested Case Control | Register | Schizophrenia | Male | Finland | 1992 | Homicide, assault, robbery, arson, violation of domestic peace | OPCRIT and clinical data used to yield DSM-III- R diagnoses | Birth cohort members with no diagnosis | DSM III-R alcohol abuse or dependence | 26 |
| Rasanen, 1998 | 0 | 25 | 10 | 5217 | Nested Case Control | Register | Schizophrenia | Female | Finland | 1992 | Homicide, assault, robbery, arson, violation of domestic peace | OPCRIT and clinical data used to yield DSM-III- R diagnoses | Birth cohort members with no diagnosis |  | na |
| Stueve,  1998 | 13 | 51 | 117 | 966 | Case-control | Self report | Psychotic/Bipolar | Male | Israel | 1992 | Self reported measures of fighting and weapon use over 5 years | Modified SADS -1 and Research Diagnostic criteria | Surveyed population without a diagnosis of psychotic/bipolar disorders/generalised anxiety disorder/phobia, non psychotic depression | Lifetime alcohol abuse, drug abuse using SADS-1 | 24-33 |
| Stueve,  1998 | 12 | 50 | 28 | 811 | Case-control | Self report | Psychotic/Bipolar | Female | Israel | 1992 | Self reported measures of fighting and weapon use over 5 years | Modified SADS -1 and Research Diagnostic criteria | Surveyed population without a diagnosis of psychotic/bipolar disorders/generalised anxiety disorder/phobia, non psychotic depression | Lifetime alcohol abuse, drug abuse using SADS-1 diagnoses | 24-33 |
| Arsenault,  2000 | 13 | 39 | 22 | 572 | Nested Case Control | Register & Self report | Schizophrenia spectrum disorder | Mixed | New Zealand | 1994 | Convictions for inciting or threatening violence, using an attack dog on a person, presenting an offensive weapon, threatening a police officer, rape, manual assault, assault on a police officer, assault with a deadly weapon, aggravated robbery and homicide. Self reported violence ascertained by interview yielding 7 item violence scale including simple assault, aggravated assault, robbery, rape and gangfighting. | DIS leading to DSM-III-R diagnoses | Birth cohort individuals with no psychiatric disorder | Marijuana dependence or alcohol dependence according to DSM-III-R | 21 |
| Brennan,  2000 | 129 | 1143 | 4421 | 163727 | Nested Case Control | Register | Schizophrenia | Male | Denmark | 1991 | Murder, attempted murder, rape, violence against authority, assault, domestic violence, robbery | ICD -8 Hospital discharge diagnoses | Birth cohort individuals without a diagnosis of schizophrenia, psychosis, personality disorder or substance misuse | ICD 8 diagnoses of drug and/or alcohol abuse | - |
| Brennan,  2000 | 19 | 680 | 151 | 150988 | Nested Case Control | Register | Schizophrenia | Female | Denmark | 1991 | Murder, attempted murder, rape, violence against authority, assault, domestic violence, robbery | ICD -8 Hospital discharge diagnoses | Birth cohort individuals without a diagnosis of schizophrenia, psychosis, personality disorder or substance misuse | ICD 8 diagnoses of drug and/or alcohol abuse | - |
| Monahan, 2000 | 13 | 160 | 24 | 519 | Longitudinal | Register, collateral and self - report | Schizophrenia | Mixed | US | 1995 | Acts of battery that resulted in physical injury, sexual assaults, assaultive acts that involved the use of a weapon, or threats made with a weapon in hand over 10 week period after discharge | Research clinician using DSM-III-R checklist | Neighbourhood community residents | DSM-III-R alcohol and drug abuse or dependence | - |
| Haller, 2001 | 51 | 1325 | 3218 | 265000 | Case-control | Register | Schizophrenia, Schizoaffective and delusional disorders | Mixed | Austria | 1997 | No definition given | ICD-10 |  | - | - |
| Wallace,  2004 | 220 | 1689 | 49 | 1689 | Case-control | Register | Schizophrenia | Male | Australia | 2000 | Offences involving interpersonal violence, including assault, causing of serious injury, and homicide but excluding threatening behaviour , property damage, sexual offences | DSM-IV diagnoses made by psychiatrist at discharge or within 1 month of first community contact | General population controls matched for age, gender and neighbourhood of residence | ‘Diagnosis of a substance related disorder or conviction for a substance related offence’ | 28-33 |
| Wallace,  2004 | 16 | 1172 | 4 | 1172 | Case-control | Register | Schizophrenia | Female | Australia | 2000 | Offences involving interpersonal violence, including assault, causing of serious injury, and homicide but excluding threatening behaviour , property damage, sexual offences | DSM-IV diagnoses made by psychiatrist at discharge or within 1 month of first community contact | General population controls matched for age, gender and neighbourhood of residence | ‘Diagnosis of a substance related disorder or conviction for a substance related offence’ | 28-33 |
| Corrigan, 2005 | 16 | 135 | 57 | 2965 | Case-control | Self report | Nonaffective disorder, psychosis and bipolar disorder | Mixed | USA | 1992 | Serious trouble with the law or physical fifth resulting in injury | DSM-III-R using CIDI | Surveyed population with no mental disorder | Abuse with or without dependence |  |
| Fazel,  2006 | 9916 | 43401 | 179536 | 3490448 | Case-control | Register | Schizophrenia & other psychoses | Male | Sweden | 2000 | Homicide, Attempted Homicide, aggravated assault, robbery, threatening behaviour, harassment, arson, any sexual crime | ICD-9/ICD-10 | General population | - | >=40 |
| Fazel,  2006 | 1583 | 54681 | 14811 | 3685913 | Case-control | Register | Schizophrenia & other psychoses | Female | Sweden | 2000 | Homicide, Attempted Homicide, aggravated assault, robbery, threatening behaviour, harassment, arson, any sexual crime | ICD-9/ICD-10 | General population | - | >=40 |
| Coid, 2006 | 6 | 25 | 260 | 2365 | Case-control | Self report | Psychosis Screen positive | Male | UK | 2000 | Self reported violence and weapon use before and after 15 years of age and in the last 5 years | 2 out of 4 criteria positive from psychosis screening questionnaire | Surveyed population without psychiatric disorder (no personality disorder, affective disorder, anxiety disorder, drug or alcohol dependence or possible psychosis) | Drug and alcohol dependence using AUDIT and SADQ | - |
| Coid , 2006 | 5 | 26 | 78 | 2603 | Case-control | Self report | Psychosis Screen positive | Female | UK | 2000 | Self reported violence and weapon use before and after 15 years of age and in the last 5 years | 2 out of 4 criteria positive from psychosis screening questionnaire | Surveyed population without psychiatric disorder (no personality disorder, affective disorder, anxiety disorder, drug or alcohol dependence or possible psychosis) | Drug and alcohol dependence using AUDIT and SADQ | - |
| Hodgins, 2007 | 4 | 10 | 2721 | 27077 | Case Control | Register | Schizophrenia & other psychoses | Male | UK | 2005 | Violence against the person, sexual offences minus prostitution related offences and robbery | Hospital inpatient diagnosis | General population born in 1953 | - | 37.2 |
| Hodgins, 2007 | 0 | 5 | 332 | 25538 | Case Control | Register | Schizophrenia & other psychoses | Female | UK | 2005 | Violence against the person, sexual offences minus prostitution related offences and robbery | Hospital inpatient diagnosis | General population born in 1953 |  |  |
| Elonheimo, 2007 | 1 | 14 | 126 | 2429 | Nested Case Control | Register | Psychotic disorder (including Schizophrenia and Schizophreniformic psychosis) | Male | Finland | 2004 | ‘Various kinds of assault and battery, and robbery’ | ICD-10 | Birth cohort members without a psychiatric disorder | Substance use disorder ICD-10 | 23 |
| Soyka, 2007 | 62 | 1662 | 936 | 100000 | Case-control | Register | Schizophrenia | Mixed | Germany | 2002 | Physical assault, aggravated assault or battery, threatening/invasion of privacy, sexual abuse of a child, robbery, attempted manslaughter, illegal restraint, involuntary manslaughter, manslaughter, murder, sexual assault, rape, to hold someone at gunpoint | ICD-9 | German general population over same years as cohort. Population violence data supplied on request from German Criminal Statistics. |  |  |
| Eriksson, 2008 | 93 | 377 | 2941 | 49021 | Nested Case Control | Register | Schizophrenia | Male | Sweden | 2005 | Homicide, manlaughter, assault, robbery, assaulting or threatening an officer, forcible confinement, rape, sexual enforcement and sexual use | ICD 7-10 | Cohort members who were not violent |  | 54.3 |
| Elbogen, 2009 | 27 | 294 | 641 | 27384 | Longitudinal | Self-report | Schizophrenia and other psychoses | Mixed | US | 2005 | Self-reported violence including using weapons in a fight, arson, forceable sex, substance-related violence, domestic violence | DSM-IV | Community sample with no mental disorder | National Institute on Alcohol Abuse and Alcoholism Alcohol Use-Disorder and Associated Disabilities Interview Schedule-DSM-IV-Version | 43 (median) |
| Fazel, 2009 | 890 | 5243 | 4003 | 52427 | Longitudinal | Register | Schizophrenia | Male | Sweden | 2006 | Homicide, Attempted Homicide, aggravated assault, robbery, illegal threats, intimidation, arson, any sexual crime | ICD 8-10 | General population matched for birth year and gender | ICD 8-10 based diagnoses but excluding those with drug-induced psychoses | 25.3 for both genders |
| Fazel, 2009 | 164 | 2760 | 273 | 27598 | Longitudinal | Register | Schizophrenia | Female | Sweden | 2006 | Homicide, Attempted Homicide, aggravated assault, robbery, illegal threats, intimidation, arson, any sexual crime | ICD 8-10 | General population matched for birth year and gender |  |  |
